# Identification of Heilongjiang crossbred beef cattle pedigrees and reveals functional genes related to economic traits based on whole-genome SNP data

**DOI:** 10.3389/fgene.2024.1435793

**Published:** 2024-07-25

**Authors:** Shuang Li, Li Liu, Zulfiqar Ahmed, Fuwen Wang, Chuzhao Lei, Fang Sun

**Affiliations:** ^1^ Key Laboratory of Combining Farming and Animal Husbandry of Ministry of Agriculture, Institute of Animal Husbandry, Heilongjiang Academy of Agricultural Sciences, Harbin, China; ^2^ College of Animal Science and Technology, Northwest A&F University, Yangling, China; ^3^ Department of Livestock and Poultry Production, Faculty of Veterinary and Animal Sciences, University of Poonch Rawalakot, Azad Kashmir, Pakistan

**Keywords:** Heilongjiang crossbred beef cattle, functional gene, genetic diversity, population genetic structure, whole genome

## Abstract

**Introduction:** To enhance the beef cattle industry, Heilongjiang Province has developed a new Crossbred beef cattle variety through crossbreeding with exotic commercial breeds. This new variety exhibits relatively excellent meat quality, and efficient reproductive performance, catering to market demands.

**Method:** This study employed whole genome resequencing technology to analyze the genetic pedigree and diversity of 19 Heilongjiang Crossbred beef cattle, alongside 59 published genomes from East Asian, Eurasian, and European taurine cattle as controls. In addition, genes related to production traits were also searched by identifying Runs of Homozygosity (ROH) islands and important fragments from ancestors.

**Results:** A total of 14,427,729 biallelic SNPs were discovered, with the majority located in intergenic and intron regions and a small percentage in exon regions, impacting protein function. Population genetic analyses including Principal Component Analysis (PCA), Neighbor-Joining (NJ) tree, and ADMIXTURE identified Angus, Holstein, and Mishima as the main ancestors of Crossbred beef cattle. In genetic diversity analysis, nucleotide diversity, linkage disequilibrium, and inbreeding coefficient analysis reveal that the genetic diversity of Crossbred beef cattle is at a moderate level, and a higher inbreeding coefficient indicates the need for careful breeding management. In addition, some genes related to economic traits are identified through the identification of Runs of Homozygosity (ROH) islands and important fragments from ancestors.

**Conclusion:** This comprehensive genomic characterization supports the targeted improvement of economically important traits in Crossbred beef cattle, facilitating advanced breeding strategies.

## 1 Background

Domesticated cattle, primarily categorized into *Bos taurus taurus* and *Bos taurus indicus*, serve as fundamental livestock for agriculture and transportation. These animals are pivotal to agricultural societies by fulfilling social demands for meat and milk production, food supply, and crop production by enhancing soil fertility through manure (Decker et al., 2014). The distribution of *B. taurus taurus* and *B. taurus indicus* is closely related to climatic conditions. *Bos taurus taurus* adapt to temperate and cold climates and are mainly distributed in the Northern Hemisphere (Buggiotti et al., 2021; Xia et al., 2023). *B. taurus indicus* have strong heat resistance and immunity, adapt to tropical and subtropical climates, and are mainly distributed around the equator and the Southern Hemisphere (Utsunomiya et al., 2019; Li et al., 2023). Recently, the research reports that domestic cattle worldwide can be categorized into five distinctly different populations by whole genome sequencing analysis: European taurine, Eurasian taurine, East Asian taurine, Chinese indicine, and Indian indicine (Chen et al., 2018). In addition, African taurine and African indicine are also very important domestic cattle populations (Pitt et al., 2019). At the same time, many studies have analyzed the genomic variation characteristics of local cattle in China through whole genome sequencing technology, such as the cold tolerance of Yanbian cattle (Shen et al., 2020), the faster growth rate and higher feed conversion rate of Jiaxian Red Cattle (Xia et al., 2021), the heat resistance and better immunity of Dianzhong cattle (Zhang et al., 2021), the heat resistance and higher meat production performance of Chaling cattle (Li et al., 2023), the higher immune performance and fine meat quality of Sanjiang cattle (Lyu et al., 2023). This provides a scientific basis for the scientific protection and genetic improvement of the genetic resources of local cattle breeds in China.

Heilongjiang Province is a major producer of dairy cattle and beef cattle (Jia et al., 2022). To promote the genetic improvement of beef cattle and accelerate the process of beef cattle breeding, based on the original varieties of cattle, pure breeds and commercial varieties such as Angus and Holstein were introduced from abroad, and new Heilongjiang Crossbred cattle varieties were formed through hybridization (Bu et al., 2021; Nie and Han, 2023). However, there are shortcomings in the development and utilization of cattle variety resources in Heilongjiang Province. Due to the excessive dependence on imported breeds and backward breeding technology, the province has no local characteristic breeds (Li et al., 2021). Therefore, it is urgent to combine advanced technologies such as genome and transcriptome with traditional breeding ideas to further promote the development of the beef cattle industry.

The Heilongjiang Crossbred cattle in this study is a breed developed to improve the beef production and quality in Heilongjiang Province. The original beef cattle with poor productivity in Heilongjiang Province were crossed with Angus to improve beef production, then crossed with Holstein to improve the reproductive ability, and finally crossed with other commercial cattle to improve beef quality. The carcass weight of Heilongjiang Crossbred cattle is more than 300 kg, the slaughter rate can reach about 55%, and the meat production is significantly improved compared with that before. At the same time, the calving age of Heilongjiang Crossbred cattle is before 2.5 years old, and the conception rate is about 80%, which has a high reproductive ability. The production performance of Heilongjiang Crossbred cattle meets the market requirements, but there is a lack of specific research on its genetic resources. To ascertain the different lineage origins and identify genes associated with economically significant traits in Heilongjiang Crossbred cattle, we conducted whole-genome resequencing on 19 individuals using the *B. taurus* reference genome assembly (ARS-UCD1.2). This process involved identifying SNPs and comparing these SNPs with those from various commercial breeds globally to assess the genetic diversity and clustering within Heilongjiang Crossbred cattle. Subsequently, we pinpointed Runs of Homozygosity (ROH) islands within the Heilongjiang Crossbred cattle genomes and identified genes implicated in meat quality and reproductive efficiency located within high-frequency ROH segments. Additionally, introgression analysis was performed to detect genes associated with valuable economic traits in the introgressed fragments of the Heilongjiang Crossbred cattle genomes. These findings provide a scientific foundation for the genetic enhancement of hybrid beef cattle, facilitating improvements in traits crucial for economic viability and productivity.

## 2 Methods

### 2.1 Samples, DNA extraction, and sequencing

Ear tissue samples of Heilongjiang Crossbred cattle (n = 19) were collected by random sampling from Huanan County, Jiamusi City, Heilongjiang Province of China. All samples are from designated farm for beef cattle molecular breeding research and genetic resource mining project. The collected samples adhered to the breed characteristics of Heilongjiang Crossbred cattle, and according to the pedigree information of the farm, there was no kinship between individuals. The Genomic DNA of the ear tissue samples was extracted using a standard phenol/chloroform-based protocol. The DNA library was constructed for each sample (500 bp insert size). The Sequencing was done via Illumina NovaSeq 6000 with a 2 × 150 bp model at Novogene Bioinformatics Institute, Beijing, China, and 150 bp paired-end sequence data were generated (the BioProject accession number PRJNA1101862). Furthermore, we obtained sequence data from 59 cattle, including East Asian taurine cattle (15 Hanwoo and 8 Mishima cattle), Eurasian taurine cattle (6 Gelbvieh and 8 Simmental cattle), and European taurine cattle (14 Angus and 8 Holstein cattle) available publicly (Supplementary Table S2). In total, 78 whole genomes of cattle were used for the subsequent analysis.

### 2.2 Reads mapping and SNP calling

The clean reads were mapped onto the *B. taurus* reference genome assembly ARS-UCD1.2 using BWA-MEM (0.7.13-r1126) with default parameters (Li and Durbin, 2009). After mapping, the SNPs were detected by using Samtools (Li et al., 2009), Picard tools (http://broadinstitute.github.io/picard), and Genome Analysis Toolkit (GATK, version 3.6-0-g89b7209). The raw SNPs were called using the “HaplotypeCaller,” “GenotypeGVCFs” and “SelectVariants” of GATK. After SNP calling, we used the “VariantFiltration” to discard sequencing and alignment artifacts from the SNPs with the parameters “QD < 2.0, FS > 60.0, MQ < 40.0, MQRankSum <−12.5, ReadPosRankSum <−8.0 and SOR > 3.0” and mean sequencing depth of variants (all individuals) “<1/3× and >3×.” By using the ANNOVAR (Wang et al., 2010), the SNPs were annotated based on the latest reference assembly (ARS-UCD1.2).

### 2.3 Population structure and phylogenetic analysis

After pruning in PLINK with the parameter (--maf 0.01 --indep-pair-wise 50 5 0.2), a set of SNPs was generated for the following analyses. Genetic distance was constructed by PLINK (--distance-matrix) and an unrooted neighbor-joining (NJ) tree was constructed based on the matrix of pairwise genetic distances using MEGA v7.0 (Kumar et al., 2016) and iTOL v5 (Letunic and Bork, 2021). The principal component analysis (PCA) was performed using the smartPCA of the EIGENSOFT v5.0 package (Patterson et al., 2006). The Population structure analysis was assessed with genetic clusters K ranging from 2 to 6 using the ADMIXTURE v1.3 (Alexander and Lange, 2011) and plotted with ggplot2 package of R.

### 2.4 Genetic diversity, linkage disequilibrium, inbreeding coefficient detection, and the number of breed-specific SNPs

We used VCFtools to estimate the nucleotide diversity of each breed in window sizes of 50 kb with 50 kb increments (Danecek et al., 2011). The Linkage disequilibrium (LD) decay with physical distance between SNPs was calculated and visualized by using PopLDdecay software with default parameters (Zhang et al., 2019). We evaluated inbreeding coefficient by proportion of the genome covered by runs of homozygosity (FROH) calculated with PLINK, which was estimated based on the total length of ROH divided by the length of autosomes per individual (McQuillan et al., 2008). The plot as mentioned above was depicted using ggplot2 package of R (R Core Team, 2023). Finally, calculate the number of breed-specific SNPs using Python (3.8.16). The idea of the Python script is to compare the SNP dataset of the target breed with the SNP datasets of other breeds, filter out duplicate SNP of the target breed and other breeds, and obtain breed specific SNP.

### 2.5 Runs of homozygosity

The runs of homozygosity (ROHs) were identified using the --homozyg option implemented in the PLINK (Purcell et al., 2007), which slides a window of 100 SNPs (-homozyg-window-snp 100 --homozyg-snp 200 --homozyg-kb 100 --homozyg-gap 1000 --homozyg-window-threshold 0.05 --homozyg-window-het 1) across the genome estimating homozygosity. In this study, we defined ROH islands by using the “- homozygous group” option in PLINK to uniformly overlap homozygous regions with frequencies higher than 0.60.

Candidate genes were obtained by annotating the identified ROH islands with gene transfer format file (ARS-UCD1.2_genomic.gtf). To better understand the gene functions and signaling pathways of the identified candidate genes, functional enrichment analysis of GO terms and KEGG pathways was performed by KOBAS 3.0 (Xie et al., 2011). The GO and KEGG pathways were considered to be significantly enriched only when the Corrected *P* value [Benjamini and Hochberg (1995)] was lower than 0.05. In addition, this study downloaded the cattle QTLs data (QTLdb_cattleARS_UCD1.gff) from the Animal QTlLdb (https://www.animalgenome.org/cgi-bin/QTLdb/index), and used Bedtools (v2.31.0) to find the QTL loci of candidate genes of ROH island related to economic traits.

### 2.6 Local ancestry inference

LOTER (Dias-Alves et al., 2018) was used to infer taurine ancestry along the genomes of Heilongjiang Crossbred cattle. We selected Angus, Holstein, and Mishima as reference panels based on the population structure. Then, the length and frequency of ancestral segments in each reference group were calculated by LOTER (Lyu et al., 2023).

### 2.7 Signatures of selection

To identify functional genes that are relevant to Heilongjiang Crossbred cattle in ancestral segments, we detected the selection signatures within Heilongjiang Crossbred cattle using the integrated haplotype score (iHS), which is calculated by selscan and then processed with 50 kb window and a step of 20 kb. We selected genes satisfying the following two conditions as candidate genes: 1) the top 1% in the ancestral segments; 2) iHS ≥ 2 and *P* value < 0.05. To better understand the gene functions and signaling pathways of the identified candidate genes, functional enrichment analysis of GO terms and KEGG pathways was performed by KOBAS 3.0 (Xie et al., 2011). The GO and KEGG pathways were considered to be significantly enriched only when the Corrected *P* value [Benjamini and Hochberg (1995)] was lower than 0.05.

## 3 Results

### 3.1 Sequencing, assembly, and identification of single nucleotide polymorphisms

The samples of 19 Heilongjiang Crossbred cattle were sequenced using whole-genome resequencing technology (Supplementary Table S1), along with 59 published whole genome sequencing data, including East Asian taurine cattle, Eurasian taurine cattle, and European taurine cattle as control (Supplementary Table S2). A total of about 665G of raw data were obtained from 19 Heilongjiang Crossbred cattle, generating 5.53 billion clean reads and aligning to the reference genome ARSUCD1.2_Btau5.0.1Y.fa with an average depth of 15.65×.

**FIGURE 1:**
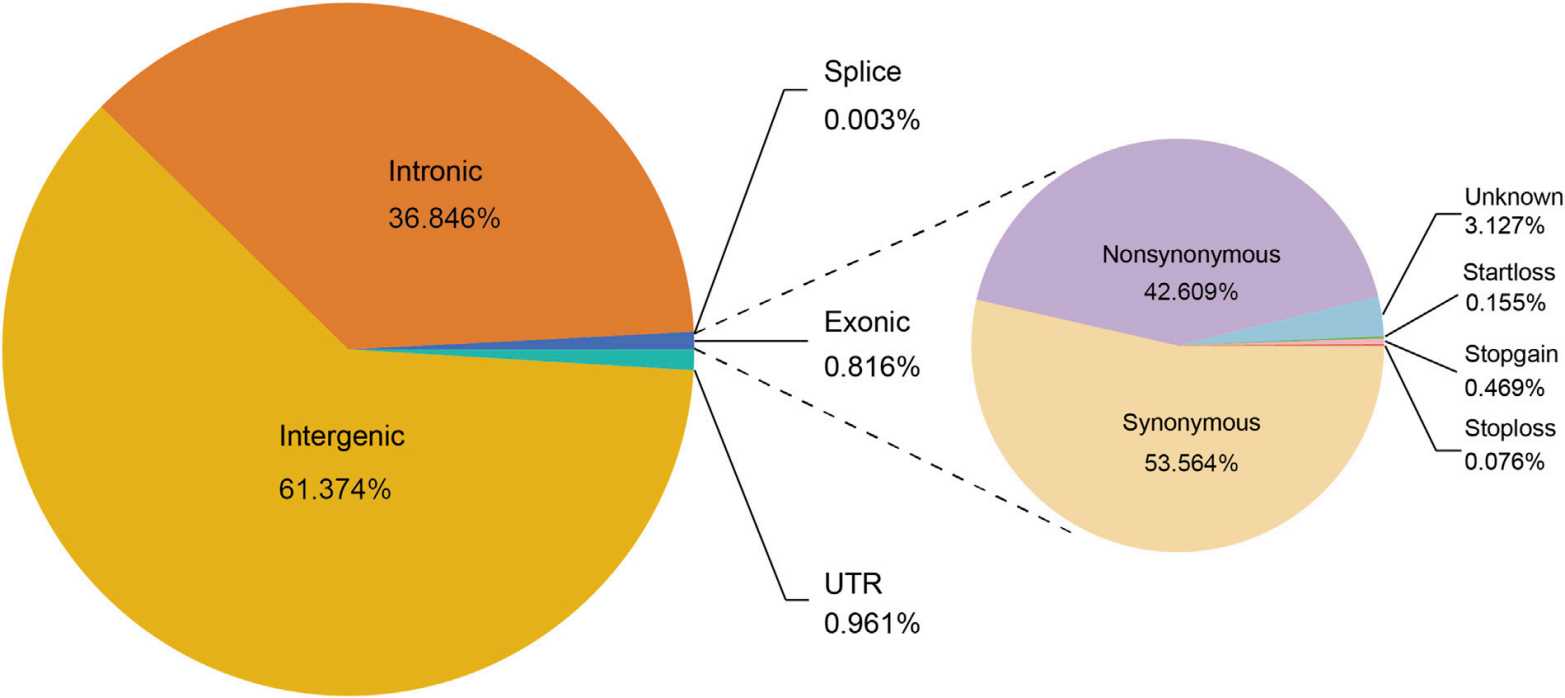
Functional classification of detected SNPs in 19 Heilongjiang Crossbred cattle genomes.

We annotated 14,427,729 biallelic SNPs that were discovered in 19 Heilongjiang Crossbred cattle. Functional annotation of the polymorphic sites revealed that 8,854,919 SNPs (61.37%) were located in the intergenic region, and 5,315,992 (36.85%) SNPs were located in the intron region. Exon accounted for 0.82% of the total SNPs, including 50,153 nonsynonymous and 63,048 synonymous SNPs (Figure 1).

### 3.2 Population structure and genetic relationships

**FIGURE 2:**
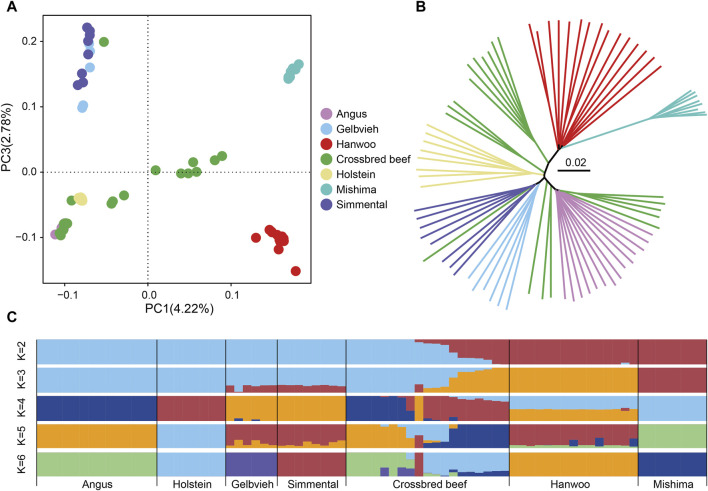
Population structure analysis of Heilongjiang Crossbred cattle. **(A)** The principal component analysis of cattle populations with PC1 against PC2. **(B)** The Neighbor-joining tree of relationships among populations. **(C)** Genetic structure of cattle populations using ADMIXTURE from K = 2 to K = 6.

To investigate the genetic relationship between Heilongjiang Crossbred cattle and other taurine cattle breeds, we conducted principal component analysis (PCA), neighbor-joining (NJ) tree, and ADMIXTURE analysis based on genomic SNPs (Figure 2). PCA shows that East Asian taurine cattle, Eurasian taurine cattle, and European taurine cattle form separate clusters. The lineage of Heilongjiang Crossbred cattle is relatively complex and scattered in distribution (Figure 2A). The NJ tree provides similar results to the above conclusions of PCA (Figure 2B). Admixture analysis revealed that when K = 6, all breeds used as reference groups are independent, and it is found that Heilongjiang Crossbred cattle are mainly of Angus and Holstein ancestry, with a small amount of Mishima ancestry (Figure 2C).

### 3.3 Population genetic diversity

**FIGURE 3:**
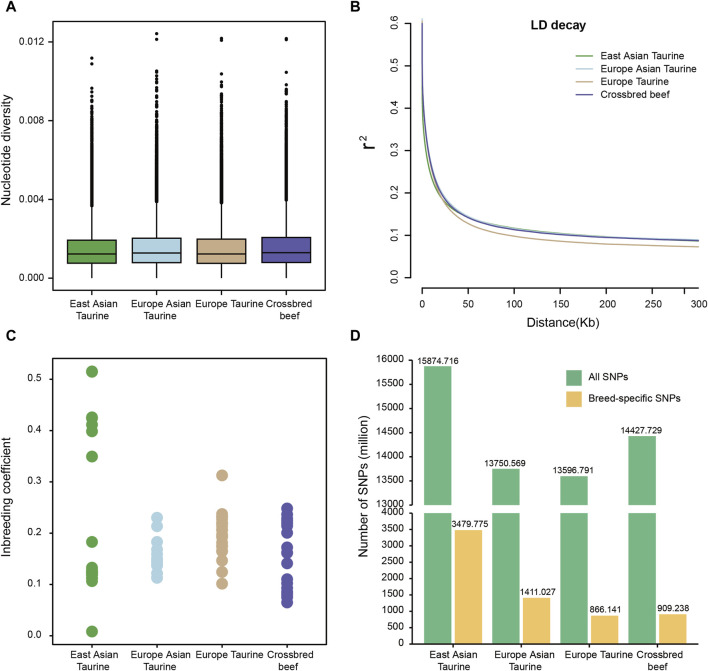
Genetic diversity among four populations. **(A)** Box plots of the nucleotide diversity for each population. The points which were on the outside of the whiskers showed outliers. **(B)** Decay of linkage disequilibrium on cattle autosomes estimated from each population. **(C)** Inbreeding coefficient for each population. **(D)** Number of SNPs for each population.

To understand the genomic diversity of Heilongjiang Crossbred cattle, we conducted nucleotide diversity analysis, LD analysis, and inbreeding coefficient analysis on four populations of East Asian taurine cattle, Eurasian taurine cattle, European taurine cattle, and Heilongjiang Crossbred cattle. As shown in Figure 3A, the nucleotide diversity of the four populations is similar, and the nucleotide diversity of Heilongjiang Crossbred cattle is slightly higher. Similarly, in the LD decay analysis, the four populations also showed a consistent trend, with little difference in the rate (Figure 3B). The analysis of the inbreeding coefficient elucidates that some East Asian taurine cattle have relatively high values for inbreeding coefficients, and there is also a certain degree of inbreeding in Heilongjiang Crossbred cattle, which may be related to the use of inbreeding methods to fix traits in the breeding process (Figure 3C; Supplementary Table S3). In addition, we also annotated the SNPs of each breed individually. There is not much difference in the number of SNPs among taurine cattle, with East Asian taurine cattle having the highest number (15,874,716). Furthermore, we found that East Asian taurine cattle possessed the highest number of specific SNPs (3,479,775), European taurine cattle possessed the lowest number of specific SNPs (866,141), and Heilongjiang Crossbred cattle possessed 909.238 specific SNPs (Figure 3D). In addition to the analysis according to the populations, we also made statistics for each breed, and the results showed that the trend of genetic diversity between breeds and populations was the same (Supplementary Figure S1). In particular, among East Asian taurine cattle, the inbreeding coefficient of Hanwoo is lower, but the inbreeding coefficient of Mishima is higher.

### 3.4 Identification and classification of ROH

**FIGURE 4:**
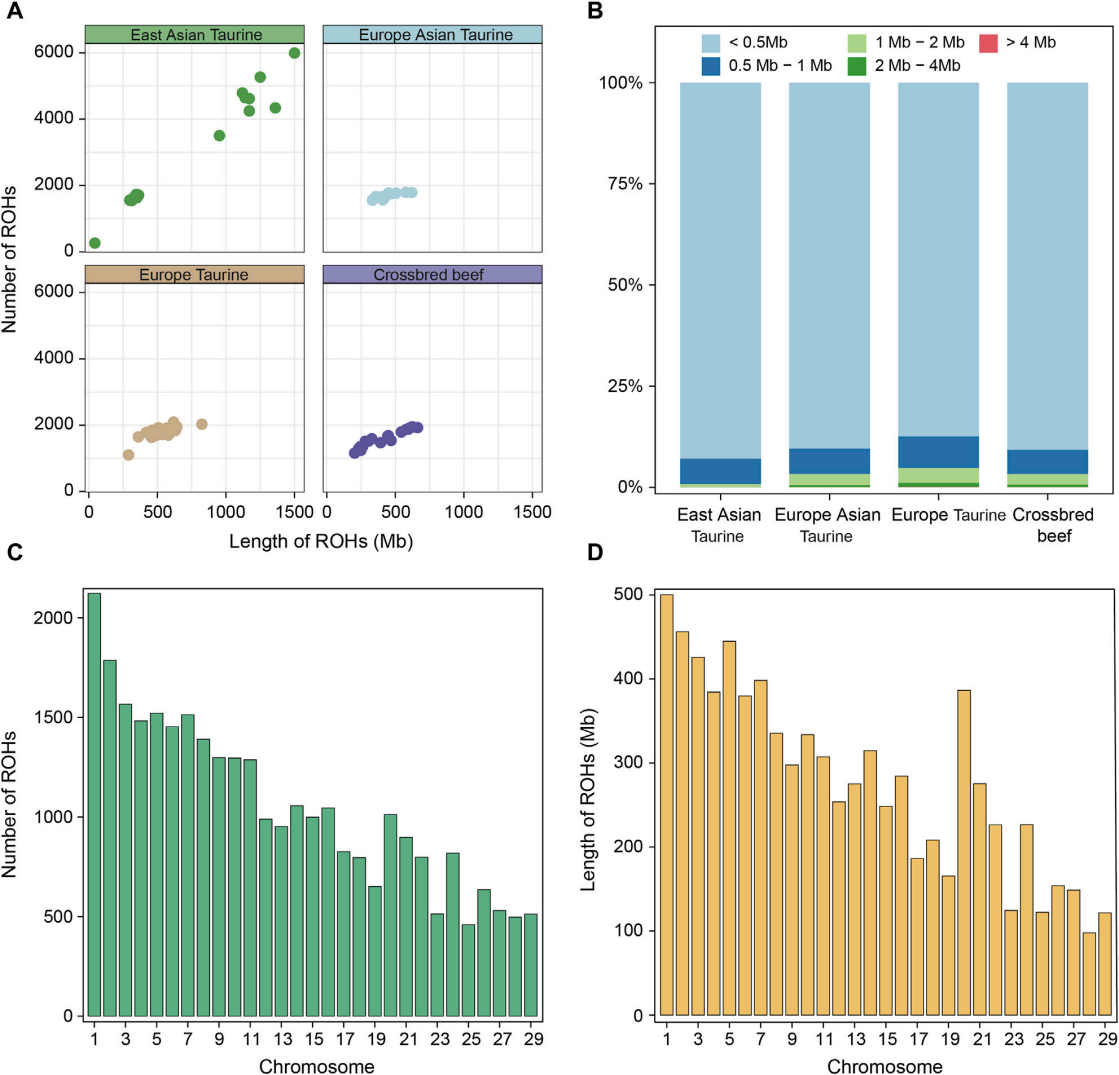
ROH analysis among four populations. **(A)** Total length and total number of ROHs per individual in each population. **(B)** The proportion of different categories of ROHs in each population. **(C)** The number of ROHs on each chromosome in Heilongjiang Crossbred cattle. **(D)** The length of ROHs on each chromosome in Heilongjiang Crossbred cattle.

Runs of homozygosity (ROHs) is a continuous homozygous region in the DNA sequence of diploid organisms. We utilized whole genome sequencing data of 78 individuals for ROH analysis. Firstly, the ROH of each individual in each population was identified and shown in Figure 4A. Except for the large difference in the length and number of ROH in East Asian taurine cattle, there was little difference between individuals in other populations. Therefore, we analyzed each breed, and the results showed that the larger difference in East Asian taurine cattle was due to the larger number and length of ROH in Mishima (Supplementary Figure S2A). After that, we divided ROH into five categories: < 0.5 Mb, 0.5–1 Mb, 1–2 Mb, 2–4 Mb, and > 4 Mb. We found that the proportion of various ROHs in each population and breed were similar, and the length of most ROHs was less than 0.5 Mb (Figure 4B; Supplementary Figure S2). Figures 4C, D show the distribution of ROH on chromosomes in Heilongjiang Crossbred cattle. It can be seen that ROH is abundantly distributed on chromosome 1 (2,123 ROHs) and least distributed on chromosome 25 (459 ROHs). In addition, ROH length is also the longest on chromosome 1 (500,103 Mb), but the shortest on chromosome 28 (97.872 Mb) (Supplementary Table S4).

### 3.5 Identification of ROH islands and gene functional annotation

**FIGURE 5:**
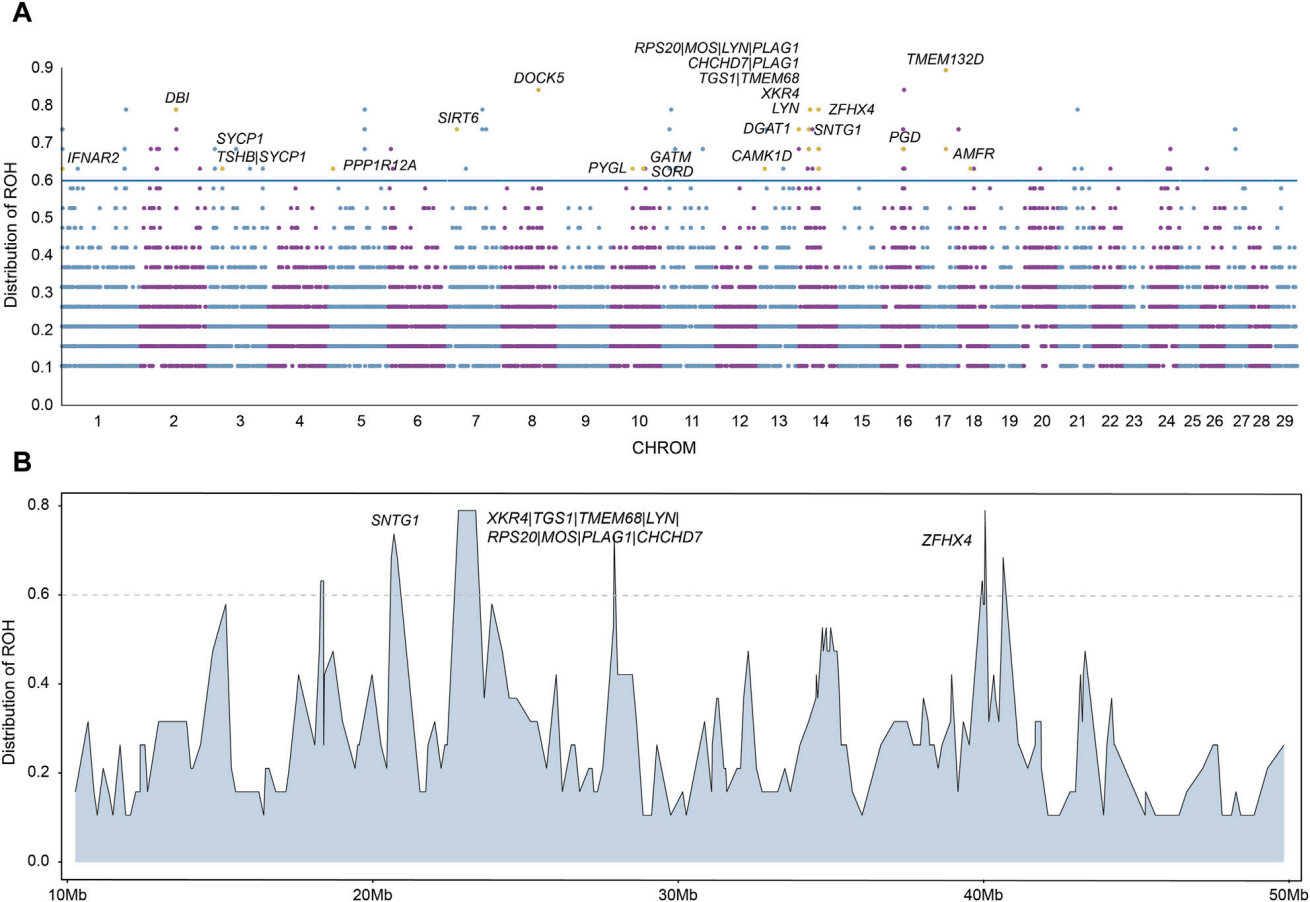
Candidate regions on ROH islands. **(A)** The distribution of ROH across autosomes. The X-axis represents the genomic coordinate, and the Y-axis displays the frequency of overlapping ROH among individuals. **(B)** Distribution of ROH on a long segment of chromosome 14.

ROH are suited to detect signatures of selection via ROH islands, we next calculated the frequency of ROH and identified ROH islands for each chromosome. The ROH frequencies are presented in Figure 5A. We regarded ROH with a frequency larger than 0.6 as ROH islands and searched for candidate genes overlapping with those ROH islands. In total, we identified 127 ROH islands. We found that the most frequent ROH island among all ROH islands is located in chromosome 17.

We annotated the 127 ROH islands identified and obtained a total of 89 candidate genes (Supplementary Table S5). We performed functional enrichment analysis using KEGG pathways and Gene Ontology (GO) for candidate genes. The results represented significant enrichment of 1 KEGG pathway term and 11 GO terms (corrected *P* value < 0.05, Supplementary Tables S6, S7). Among these 89 candidate genes with important functions were found, including reproduction (*AMFR, CAMK1D, GATM, IFNAR2, PGD, PYGL, SORD, SYCP1, TMEM132D*), growth (*MOS, PLAG1, TSHB, ZFHX4*) and meat quality (*CHCHD7, DBI, DGAT1, DOCK5, LYN, PPP1R12A, RPS20, SIRT6, SNTG1, TGS1, TMEM68, XKR4*) (Table 1). In particular, in chromosome 14, a long segment annotated with multiple genes is found (Figure 5B). In order to verify that these genes are indeed related to economic traits, ROH islands containing candidate genes were compared with cattle QTLs data (QTLdb_cattleARS_UCD1.gff), and the results showed that these genes do have QTLs related to economic traits (Supplementary Table S8).

**TABLE 1:** Candidate genes on ROH islands.

Chr	Candidate genes	ROH frequency	Trait and references
1	IFNAR2	0.6316	Bovine placental development; pregnancy maintenance (Wang et al., 2018; Wang et al., 2022)
2	DBI	0.7895	Regulates fat content (Ramírez-Zamudio et al., 2023)
3	SYCP1	0.6316	Meiotic chromosome synapses (Billmyre et al., 2023)
3	TSHB	0.6316	Growth traits (Yang et al., 2022)
5	PPP1R12A	0.6316	Beef quality traits (Sun et al., 2013; Choi et al., 2015; Xia et al., 2021)
7	SIRT6	0.7368	Fat content (Gui et al., 2018)
8	DOCK5	0.8421	Lumbar muscle area (Zhao et al., 2024)
10	GATM	0.6316	Follicular overdevelopment (Bunel et al., 2014)
10	PYGL	0.6316	Embryonic development (Banliat et al., 2020)
10	SORD	0.6316	Reproductive system-related functions (Johnston et al., 2018)
13	CAMK1D	0.6316	Heifer early calving (Costa et al., 2015; Melo A et al., 2018; Sbardella et al., 2021)
14	CHCHD7	0.7895	Carcass weight (Nishimura et al., 2012; Randhawa et al., 2014)
14	DGAT1	0.7368	TAG synthesis and fat metabolism (Khan et al., 2021)
14	LYN	0.7895	Carcass weight (Ghoreishifar et al., 2020; Mota et al., 2022; Alam et al., 2023)
14	MOS	0.7895	Energy metabolism and feeding control (Mota et al., 2022)
14	PLAG1	0.7895	Carcass (Bejarano et al., 2023; Hu et al., 2023; Hu et al., 2024)
14	RPS20	0.7895	Back fat thickness and hip fat thickness (Medeiros de Oliveira Silva et al., 2017; Alam et al., 2023)
14	SNTG1	0.7368	Body length traits (An et al., 2019)
14	TGS1	0.7895	Carcass and meat quality traits (Ramayo-Caldas et al., 2014)
14	TMEM68	0.7895	Lipid biosynthesis (Lindholm-Perry et al., 2012)
14	XKR4	0.7895	Lipid metabolism; meat quality (Lindholm-Perry et al., 2012; Alam et al., 2023; Arikawa et al., 2024)
14	ZFHX4	0.7895	Pulsatile release of GnRH (Fortes et al., 2011)
16	PGD	0.6842	Early pregnancy (Johnston et al., 2018)
17	TMEM132D	0.8947	Reproductive traits (Zhang et al., 2019)
18	AMFR	0.6316	Embryonic development (Légaré et al., 2017)

### 3.6 Local ancestry inference of Heilongjiang crossbred cattle and selection signatures with different ancestry

**FIGURE 6:**
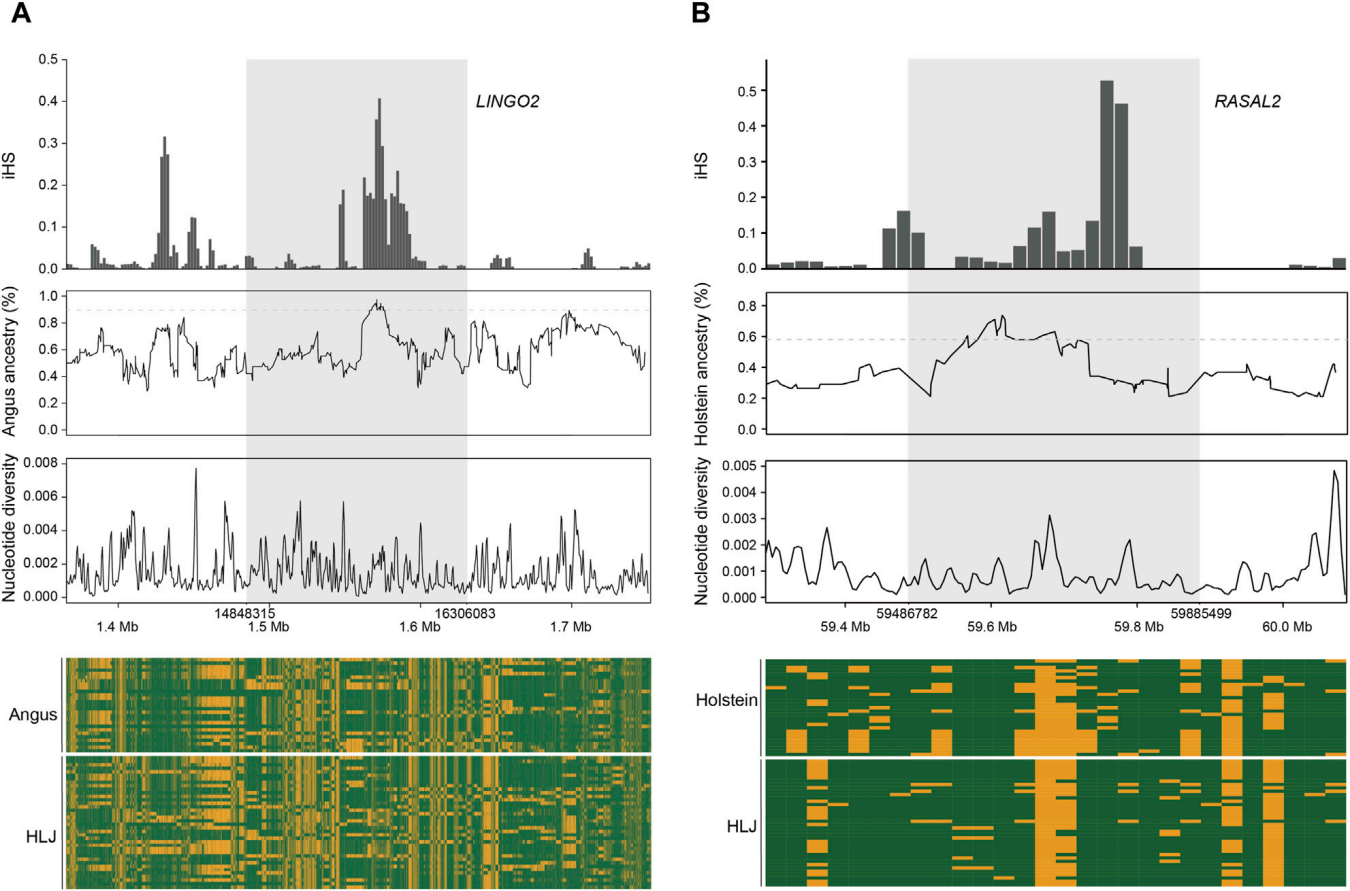
Example of candidate selective loci with taurine ancestry. **(A)** *LINGO2* gene. **(B)** *RASAL2* gene.

We used LOTER to infer the taurine cattle ancestry in the Heilongjiang Crossbred cattle genome (Dias-Alves et al., 2018). According to the previous population structure analysis, Angus, Holstein, and Mishima were selected as the reference group. The results showed that Angus cattle had the highest infiltration frequency, followed by Holstein, and Mishima had the lowest infiltration frequency. To screen the ancestral segments that are important for Heilongjiang Crossbred cattle, we detected the selection signatures within Heilongjiang Crossbred cattle using an integrated haplotype score (iHS). Finally, the fragments with the top 1% introgression frequency overlapping with the selected fragments in the iHS method (iHS ≥ 2 and *P* value < 0.05) were considered high-frequency ancestral fragments. After that, these fragments were annotated, resulting in 165 (Angus), 90 (Holstein), and 103 (Mishima) genes, respectively (Supplementary Tables S9–S11). Among these genes, we found some genes related to the economic traits of Heilongjiang Crossbred cattle (Figure 6), including reproductive traits (*BCAR3*, *CDH13*, *CDH18*, *KMT2C*, *MACROD2*, *PCSK5*, *PPP1R1C*, *SCAPER*, *ZPBP*) and meat production traits (*AGBL4*, *ARFGAP3*, *AUTS2*, *DPH6*, *DPP6*, *EEPD1*, *GRID2*, *LEKR1*, *LINGO2, MAP3K5*, *NEGR1*, *NKAIN2*, *PLCD1*, *PRKN*, *RASAL2*, *RHOBTB1*, *TRIM64*, *TRIM77*, *WWOX*) (Table 2), reflecting the role of ancestral segments for Heilongjiang Crossbred cattle.

**TABLE 2:** Candidate genes with taurine ancestry.

Chr	Candidate genes	Ancestral lineages (introgression frequency)	iHS	Trait and references
1	LEKR1	Angus (0.9211)	3.18	Morphology; growth traits (Ceccobelli et al., 2023)
2	PPP1R1C	Holstein (0.6316)	3.06	Litter size (Mahmoudi et al., 2022)
3	AGBL4	Angus (0.5789)	4.50	Fat metabolism (Akanno et al., 2018)
3	BCAR3	Angus (0.9737)	5.16	Endometrium (Meng et al., 2019)
3	NEGR1	Holstein (0.6842)	4.13	Fat metabolism (Faggion et al., 2023)
4	DPP6	Mishima (0.4474)	4.49	Average daily weight gain (Sheet et al., 2024)
4	EEPD1	Holstein (0.6842)	2.93	Fat metabolism (Kunath et al., 2016; Xu et al., 2022)
4	KMT2C	Holstein (0.5789)	3.42	Spermatogenesis (Khan et al., 2021)
4	ZPBP	Angus (0.8947)	3.09	Spermatogenesis (Gao et al., 2019)
5	ARFGAP3	Angus (0.8947)	4.74	Rib eye area (Santana et al., 2015; Taye et al., 2017)
6	GRID2	Angus (0.9737)	4.57	Growth (Peng et al., 2024)
8	LINGO2	Angus (0.9737)	4.08	Meat quality; obesity (Williams et al., 2012; An et al., 2020; Lyu et al., 2023)
8	PCSK5	Angus (0.8947)	3.83	Fertility (Chen et al., 2022)
9	MAP3K5	Mishima (0.3947)	2.86	Muscle structure and metabolism (Taye et al., 2017)
9	NKAIN2	Mishima (0.4211)	3.69	Body mass index; total dietary fat intake; growth; fat development (Rudkowska et al., 2015; Yasukochi et al., 2018; Ding et al., 2022)
9	PRKN	Angus (0.8947)	4.00	Fat metabolism (Wang et al., 2019; Jourshari et al., 2023)
10	DPH6	Angus (0.9211)	3.90	Fat deposition (Martins et al., 2021)
13	MACROD2	Angus (0.8947)	3.93	Semen quality (Ebenezer Samuel King et al., 2022)
16	RASAL2	Holstein (0.7368)	4.25	Weight gain; lipogenesis (Thorleifsson et al., 2009; Zhu et al., 2017)
18	CDH13	Holstein (0.8947)	4.05	Early reproductive development (Coen et al., 2023)
18	WWOX	Angus (0.8947)	3.49	Backfat thickness (Arikawa et al., 2024)
20	CDH18	Mishima (0.4474)	4.55	Primiparity age (Ahmad et al., 2023)
21	SCAPER	Angus (0.9737)	4.14	Spermatogenesis (Tatour et al., 2020; Ghoreishifar et al., 2023)
22	PLCD1	Angus (0.9474)	3.85	Fat metabolism (Taye et al., 2017)
25	AUTS2	Holstein (0.7632)	3.40	Feed efficiency (Beunders et al., 2013; Beunders et al., 2015)
28	RHOBTB1	Angus (0.9474)	3.34	Rib eye area (Silva et al., 2020)
29	TRIM64	Angus (0.8947)	3.44	Backfat thickness (Wu et al., 2023)
30	TRIM77	Angus (0.9211)	2.34	Backfat thickness (Grigoletto et al., 2020)

## 4 Discussion

As living standards have risen, there has been a corresponding increase in consumer demand for beef and dairy products in recent years. Heilongjiang Province, serving as a principal production hub for dairy and beef cattle, has hybridized native breeds with commercially purebred cattle to develop a new crossbred variety. This innovation aims to satisfy the escalating consumer demand for beef. However, at present, there is little understanding of the genetic resource of Heilongjiang Crossbred cattle, so it is of great significance to study the genetic diversity and population structure of Heilongjiang Crossbred cattle through whole genome sequencing data.

We resequenced the whole genome of 19 Heilongjiang Crossbred cattle and selected possible ancestors: East Asian taurine cattle, Eurasian taurine cattle, and European taurine cattle as reference populations for comparative analysis. We annotated the final generated SNP set, and the results showed that most SNPs were located in intergenic regions, and SNPs located in exons accounted for only 0.82% of the total SNPs, which was consistent with previous studies (Shen et al., 2020; Xia et al., 2021; Lyu et al., 2023). Among the SNPs located in exons, 42.61% were nonsynonymous SNPs and 53.56% were synonymous SNPs (Figure 1).

Next, we used the whole genome data of 78 individuals to analyze the genetic relationship between Heilongjiang Crossbred cattle and other breeds. We performed PCA, NJ tree, and ADMIXTURE based on genomic SNPs. The results of PCA and NJ tree are similar. East Asian taurine cattle, Eurasian taurine cattle, and European taurine cattle form independent clusters, which play a reference role. Heilongjiang Crossbred cattle are scattered among different breeds, representing the existence of hybridization of different lineages, which are more complex. In the ADMIXTURE, the CV value is the smallest when K = 2, and it is evident that Heilongjiang Crossbred cattle have lineage hybridization. But when K = 2, the specific bloodline composition of Heilongjiang hybrid cattle is not obvious. When K = 6, all breeds used as the reference population are independent. At this time, it can be seen that the Heilongjiang Crossbred cattle are mainly of Angus and Holstein ancestry, followed by the Mishima ancestry, and a very small number of Gelbvieh and Simmental ancestry. PCA, NJ tree and ADMIXTURE analysis all proved that Heilongjiang Crossbred cattle came from the hybridization of different lineages.

Then the SNP data set of 78 individuals was used to analyze the genetic diversity of Heilongjiang Crossbred cattle. Because the four populations belong to taurine, the genetic diversity is not different. Among them, the nucleotide diversity of Heilongjiang Crossbred cattle was slightly higher (Figure 3A), the LD decay rate was slightly faster (Figure 3B), and the genetic diversity was relatively high. According to the inbreeding coefficient analysis of each individual, it was found that the inbreeding degree of some individuals in East Asian taurine cattle was relatively high, and there was also a certain degree of inbreeding in Heilongjiang Crossbred cattle (Figure 3C), which may be related to the inbreeding method adopted in the breeding process of Heilongjiang Crossbred cattle, and the degree of inbreeding needs to be properly controlled in the subsequent breeding process. In addition, the total number of SNPs and the number of specific SNPs in each population were also counted. The results showed that the four populations had little difference, and the Heilongjiang Crossbred cattle belonged to the medium level (Figure 3D), which was consistent with the results of genetic diversity. At the same time, we counted the number of SNPs of each breed separately (Supplementary Figure S1), and the results showed that the number of SNPs of Heilongjiang Crossbred cattle was higher than that of each ancestral source, which may be due to the heterosis generated by hybridization.

In addition, we also conducted a systematic analysis of the ROHs of 78 individuals. The presence of long ROHs results from inbreeding, while shorter ROHs reflect the influence of ancient ancestors (Purfield et al., 2012). First, we compared the total length and total number of ROHs of each individual in each population (Figure 4A), and the results showed that except for the large individual differences in East Asian taurine cattle, the ROHs of the other three populations were similar in number and length. The large individual differences in East Asian taurine cattle are due to the large difference between Hanwoo and Mishima (Supplementary Figure S2), which may be due to the high degree of artificial selection of Mishima. After that, the ROHs of each population were divided into five categories according to < 0.5 Mb, 0.5–1 Mb, 1–2 Mb, 2–4 Mb, and > 4 Mb, and the proportion of different categories of ROHs in each population was shown (Figure 4B). The results showed that the length with the largest proportion in each population was <0.5 Mb, but the number of long-segment ROHs of East Asian taurine cattle was the least, and the number of long-segment ROHs of European taurine cattle was the most. The long segment ROHs of Heilongjiang Crossbred cattle belonged to the medium level in the four populations, indicating that there was a certain degree of hybridization in Heilongjiang Crossbred cattle, which was consistent with the results of the inbreeding coefficient. Finally, we counted the number and length of ROHs in each chromosome of Heilongjiang Crossbred cattle (Figures 4C, D). The number of ROHs on the chromosome is the largest (2, 123 ROHs) and the length is the longest (500, 103 Mb). The lowest number of ROHs was on chromosome 25 (459 ROHs), followed by chromosome 28 (497 ROHs) and chromosome 29 (512 ROHs). The shortest length of ROHs is chromosome 28 (97.872 Mb), followed by chromosome 29 (121.593 Mb) and chromosome 25 (122.323 Mb). The trend of the number and length distribution of ROHs on all chromosomes was consistent with previous studies (Zhao, 2021).

In addition to the statistics of ROHs of each population, we also identified ROH islands of hybrid beef cattle and detected some genes related to economic traits. We regarded ROHs with a frequency greater than 0.6 in Heilongjiang Crossbred cattle as ROH islands, and a total of 127 ROH islands were identified. These ROH islands were annotated and a total of 89 candidate genes were obtained (Supplementary Table S5). We performed functional enrichment analysis using KEGG pathways and Gene Ontology (GO) for candidate genes. The results represented significant enrichment of 1 KEGG pathway term and 11 GO terms (corrected *P*-value < 0.05, Supplementary Tables S6, S7). Among these 89 candidate genes, we found some genes with important functions (Figure 5A), including reproduction (*AMFR, CAMK1D, GATM, IFNAR2, PGD, PYGL, SORD, SYCP1, TMEM132D*), growth (*MOS, PLAG1, TSHB, ZFHX4*) and meat quality (*CHCHD7, DBI, DGAT1, DOCK5, LYN, PPP1R12A, RPS20, SIRT6, SNTG1, TGS1, TMEM68, XKR4*). Comparing these genes with cattle QTLs data, the results also proved that these genes were related to economic traits (Supplementary Table S8).

Heilongjiang Crossbred cattle are bred to meet the growing demand of people, and have good reproductive ability and meat production ability. Therefore, some genes related to reproductive ability have also been found in ROH Islands (*AMFR, CAMK1D, GATM, IFNAR2, PGD, PYGL, SORD, SYCP1, TMEM132D*). *AMFR* and *PYGL* play important roles in embryonic development (Légaré et al., 2017; Banliat et al., 2020). *CAMK1D, GATM*, *IFNAR2,* and *PGD* have effects on the reproductive function of cows and affect the reproductive function of cows from different aspects. *CAMK1D* was found to be associated with heifer early calving until 30 months and stability traits in Nelore cattle (Costa et al., 2015; Melo A et al., 2018; Sbardella et al., 2021). *GATM* is associated with bovine follicular overdevelopment, affecting follicular function and oocyte quality (Bunel et al., 2014). *IFNAR2* plays an important role in bovine placental development and pregnancy maintenance (Wang et al., 2018; Wang et al., 2022). *PGD* is a potential marker of early pregnancy (Johnston et al., 2018). In addition, *SORD* exerts reproductive system-related functions and affects the reproductive ability of bulls (Johnston et al., 2018). *SYCP1* is essential for meiotic chromosome synapses *in vivo* and plays an important role in sexual reproduction (Billmyre et al., 2023). *TMEM132D* is a candidate gene that constitutes the potential genetic structure of porcine reproductive traits (Zhang et al., 2019). It is also selected in Heilongjiang Crossbred cattle, and it is presumed to have the same effect on the reproductive traits of cattle. These genes play roles in reproductive traits from different aspects, which may be the reason why Heilongjiang Crossbred cattle have higher reproductive efficiency.

In addition to reproductive traits, the functional genes identified in ROH islands also have genes related to meat production ability, including growth (*MOS, PLAG1, TSHB, ZFHX4*) and meat quality (*CHCHD7, DBI, DGAT1, DOCK5, LYN, PPP1R12A, RPS20, SIRT6, SNTG1, TGS1, TMEM68, XKR4*). Four genes were identified in terms of growth traits. *MOS* has an impact on energy metabolism and feeding control, affecting the growth rate of beef cattle by affecting feed efficiency (Mota et al., 2022). *IGF-2* is a cell growth and differentiation factor that plays an important role in muscle growth and differentiation in cattle (Huang et al., 2014; Júnior et al., 2016), *PLAG1* regulates *IGF-2* expression (Van Dyck et al., 2007; Akhtar et al., 2012; Utsunomiya et al., 2017). Therefore, *PLAG1* plays an important role in carcass and has been selected in many studies on cattle (Bejarano et al., 2023; Hu et al., 2023; Hu et al., 2024). *TSHB* was identified to be associated with growth traits in chickens (Yang et al., 2022). It was also screened in this study, so we speculated that it would play a similar role in Cross-bred beef cattle. *ZFHX4* regulates the onset of publication by affecting the pulsatile release of GnRH, thereby promoting beef cattle growth (Fortes et al., 2011). A total of 12 genes were identified in meat production traits, which can be divided into two aspects. The first is fat synthesis and fat metabolism. Intramuscular fat, which determines the tenderness and flavor of beef to a certain extent, is an important part of beef production (Hunt et al., 2014). *DBI* regulates fat content by upregulating the PPAR signaling pathway (Gross et al., 2017; Chen et al., 2022; Ramírez-Zamudio et al., 2023). Triacylglycerol (TAG) is the main component of intramuscular fat, and *DGAT1* affects intramuscular fat content by participating in tag synthesis and fat metabolism (Khan et al., 2021). *PPP1R12A* is one of the genes highly expressed in porcine intramuscular adipose tissue and has been selected in other studies on beef quality traits (Sun et al., 2013; Choi et al., 2015; Xia et al., 2021). *SIRT6* and *TGS1* regulate fat content and meat quality traits by affecting transcript levels (Ramayo-Caldas et al., 2014; Gui et al., 2018). *TMEM68* is expressed in rumen, abdominal stomach, intestine and adipose tissue of cattle and may affect lipid biosynthesis (Lindholm-Perry et al., 2012). The protein encoded by *XKR4* plays an important biological role in cellular and lipid metabolism (Lindholm-Perry et al., 2012), and has been reported in several genes related to meat quality traits (Alam et al., 2023; Arikawa et al., 2024). These genes affect the intramuscular fat content of Heilongjiang Crossbred cattle from different pathways and play an important role in meat quality. The second part is the genes that affect other meat production indicators of beef cattle. *CHCHD7* has been strongly selected in studies on bovine body size (Randhawa et al., 2014), and was considered one of the three major QTLs for carcass weight in Japanese black cattle (Nishimura et al., 2012). *DOCK5* was identified to be associated with the lumbar muscle area (Zhao et al., 2024). *LYN* is related to the carcass weight of beef cattle in several studies (Ghoreishifar et al., 2020; Mota et al., 2022; Alam et al., 2023). *RPS20* is the reason for the difference in back fat thickness and hip fat thickness in Nelore cattle (Medeiros de Oliveira Silva et al., 2017), and was selected in the study of carcass traits of Korean Hanwoo Cattle (Alam et al., 2023). Genome-wide association study reveals that *SNTG1* is associated with increasing body length traits in Chinese Wagyu beef cattle (An et al., 2019). These genes affected the body size data and carcass weight of Heilongjiang Crossbred cattle and improved the meat production of Heilongjiang Crossbred cattle.

In particular, in chromosome 14, a long segment annotated with multiple genes is found (Figure 5B). This fragment contains a total of 10 genes, which affect Heilongjiang Crossbred cattle from different aspects so that Heilongjiang Crossbred cattle can produce more high-quality beef.

Through the analysis of population structure, we found that the Heilongjiang Crossbred cattle were mainly of Angus, Holstein, and Mishima pedigrees. To study the influence of ancestral lineage on Heilongjiang Crossbred cattle, we calculated the introgression proportion of the three ancestral lineages with LOTER. The results showed that Angus had the highest penetration frequency, followed by Holstein, and Mishima had the lowest penetration frequency, which was consistent with the results of the population structure analysis. To screen the ancestral segments that have important effects on Heilongjiang Crossbred cattle, we combined the iHS method to detect the selection signatures within Heilongjiang Crossbred cattle. Finally, the fragments with the top 1% introgression frequency overlapped with the selected fragments in the iHS method (iHS ≥ 2, *P* value < 0.05), which were considered to be high-frequency ancestral fragments with important roles. These fragments were then annotated, resulting in 165 (Angus), 90 (Holstein), and 103 (Mishima) genes, respectively (Supplementary Tables S9–S11). Among these genes, we found some genes related to economic traits of Heilongjiang Crossbred cattle (Figure 6), including reproductive traits (*BCAR3*, *CDH13*, *CDH18*, *KMT2C*, *MACROD2*, *PCSK5*, *PPP1R1C*, *SCAPER*, *ZPBP*) and meat production traits (*AGBL4*, *ARFGAP3*, *AUTS2*, *DPH6*, *DPP6*, *EEPD1*, *GRID2*, *LEKR1*, *LINGO2*, *MAP3K5*, *NEGR1*, *NKAIN2*, *PLCD1*, *PRKN*, *RASAL2*, *RHOBTB1*, *TRIM64*, *TRIM77*, *WWOX*). These genes reflect the role of high-frequency ancestral segments for Heilongjiang Crossbred cattle.

Among the genes related to reproductive traits, five genes are related to male reproduction, three genes are related to female reproduction, and the remaining one is related to embryonic development. *CDH13* plays an important role in the early reproductive development of bulls (Coen et al., 2023). *MACROD2* variation was associated with semen quality and was a biomarker of bull semen quality (Ebenezer Samuel King et al., 2022). *SCAPER* is highly expressed in testis and found to be involved in spermatogenesis in multiple species (Tatour et al., 2020; Ghoreishifar et al., 2023). *KMT2C* and *ZPBP* were identified as target genes of bull sexual maturation and associated with spermatogenesis (Gao et al., 2019; Khan et al., 2021). These five genes improve the fertility of bulls from three aspects: hormone regulation, semen quality, and spermatogenesis. *BCAR3* is expressed in the endometrium (Meng et al., 2019), the resulting variation is related to the number of stillborn pigs and was selected in the study on Nellore cattle (Wang et al., 2022; Ahmad et al., 2023). *CDH18* and *PCSK5* are related to the age of first calving and the fertility of Holstein cattle, respectively (Chen et al., 2022; Ahmad et al., 2023). While *PPP1R1C* was reported to be associated with litter size (Mahmoudi et al., 2022).

Genes that affect carcass quality fat production and metabolism are crucial for optimizing the genetic selection process of beef cattle. We also found some genes related to meat quality traits in the introgressed genes. *AGBL4*, *EEPD1*, *NEGR1*, *PLCD1* and *PRKN* are all related to fat metabolism and affect beef quality by affecting intramuscular fat content (Kunath et al., 2016; Taye et al., 2017; Akanno et al., 2018; Wang et al., 2019; Xu et al., 2022; Faggion et al., 2023; Jourshari et al., 2023). *DPH6*, *LINGO2*, *NKAIN2*, and *RASAL2* are related to weight gain and fat generation and development, and affect meat quality by affecting fat deposition (Thorleifsson et al., 2009; Williams et al., 2012; Rudkowska et al., 2015; Zhu et al., 2017; Yasukochi et al., 2018; An et al., 2020; Martins et al., 2021; Ding et al., 2022; Lyu et al., 2023). *MAP3K5* affects meat tenderness by participating in muscle structure and metabolism (Taye et al., 2017). These genes may be the reason for the delicate meat quality of Heilongjiang Crossbred cattle.

In addition, there are some genes related to meat production. *ARFGAP3* and *RHOBTB1* are related to the rib eye area (REA) of Nellore cattle and have been selected in other studies on beef cattle (Santana et al., 2015; Taye et al., 2017; Silva et al., 2020). *AUTS2* is associated with digestive system diseases (Beunders et al., 2013; Beunders et al., 2015), affecting the feed efficiency of cattle and thus growth speed (Hu et al., 2023). *DPP6* is a candidate gene for average daily weight gain (ADG), which is associated with the growth rate of cattle (Sheet et al., 2024). In the study of sheep, *LEKR1* was found to be related to morphology and growth traits (Ceccobelli et al., 2023), and we speculated that it has a similar role in beef cattle. Meanwhile, *GRID2* was also identified to be associated with growth (Peng et al., 2024). *TRIM64*, *TRIM77,* and *WWOX* were detected as candidate genes for backfat thickness (BFT) (Grigoletto et al., 2020; Wu et al., 2023; Arikawa et al., 2024). These genes help Heilongjiang Crossbred cattle grow rapidly to produce more beef.

Among the numerous genes related to economic traits, we selected two for display (Figure 6). The higher iHS frequency and lower nucleotide diversity represent that genes are selected and fixed in Heilongjiang Crossbred cattle. According to the introgression frequency of the ancestral fragment and the haplotype heatmap, it can be seen that the gene is an introgression fragment from the ancestor and the haplotype of Heilongjiang Crossbred cattle is the same as that of the ancestor. Therefore, these two genes are thought to be derived from the ancestral introgression and fixed in Heilongjiang Crossbred cattle. They play an important role in the economic traits of Heilongjiang Crossbred cattle: improving the reproductive efficiency of Heilongjiang Crossbred cattle and making Heilongjiang Crossbred cattle produce better quality beef.

## 5 Conclusion

In summary, our study utilized whole genome sequencing to explore the population structure of Heilongjiang Crossbred cattle, elucidate their genetic diversity, and identify genes related to economic traits through ROH islands and ancestral infiltration fragments. The results showed that Heilongjiang Crossbred cattle are mainly of Angus ancestry, followed by Holstein ancestry, and a small amount of Mishima ancestry with modest genetic diversity. In addition, we identified key candidate genes related to reproductive efficiency and beef quality through ROH islands and high-frequency ancestral infiltration fragments, providing new insights into the genetic basis of higher reproductive efficiency and stronger meat production ability in Heilongjiang Crossbred cattle. These findings not only enhance our understanding of the unique characteristics of Heilongjiang Crossbred cattle but also have significance in promoting crossbreeding improvement in beef cattle. However, the number of populations in this study is relatively small, and the number of populations can be expanded for more detailed research in the future.

## Data Availability

The datasets presented in this study can be found in online repositories. The names of the repository/repositories and accession number(s) can be found in the article/Supplementary Material.
