# Supplementary figures and images for: Identification of Heilongjiang crossbred beef cattle pedigrees and reveals functional genes related to economic traits based on whole-genome SNP data
Source: Front Genet. 2024 Jul 25;15:1435793. doi: 10.3389/fgene.2024.1435793 (PMC11306169; doi:10.3389/fgene.2024.1435793)

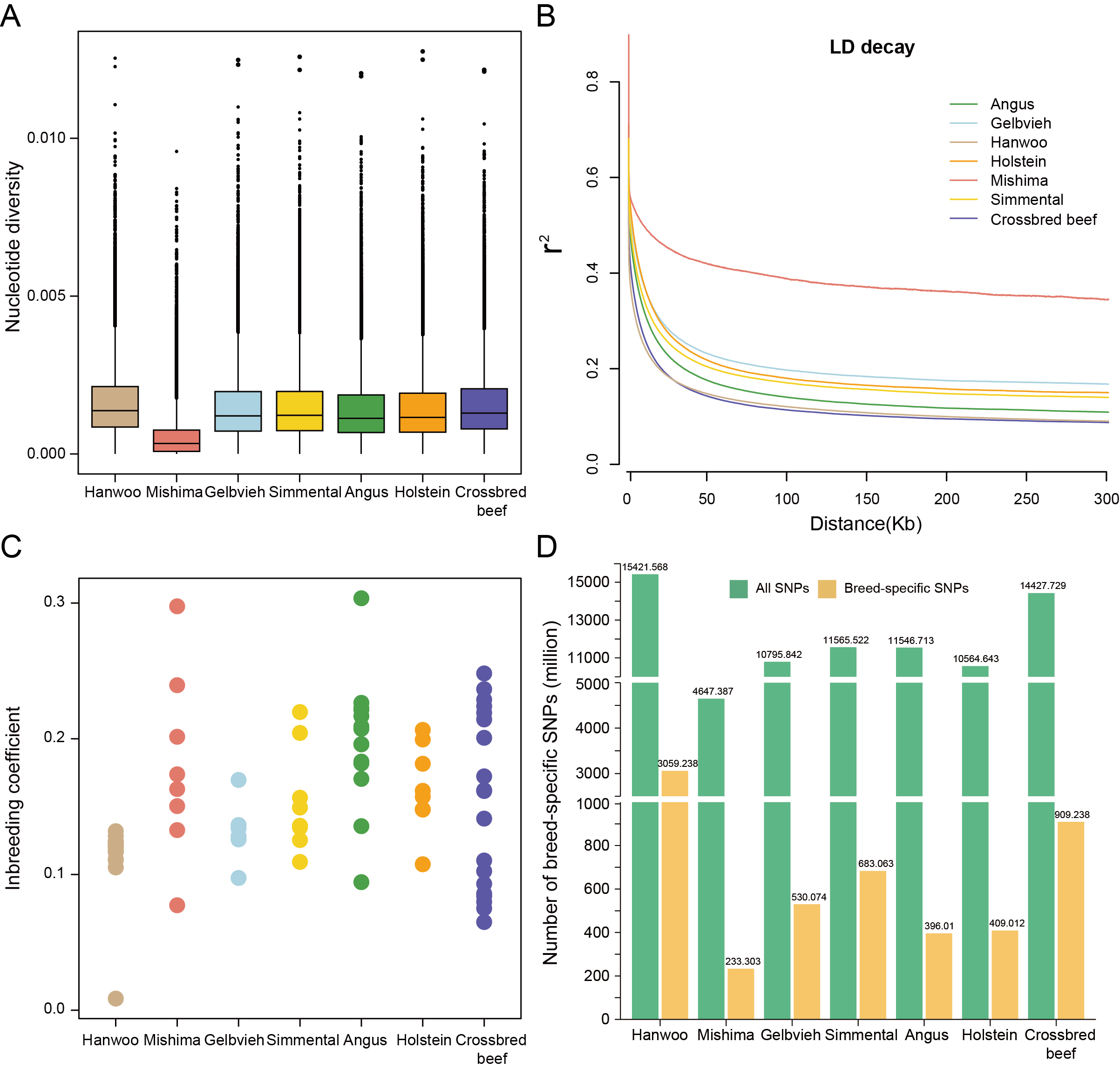

Supplement: Supplementary file 2 [file Image1.JPEG]

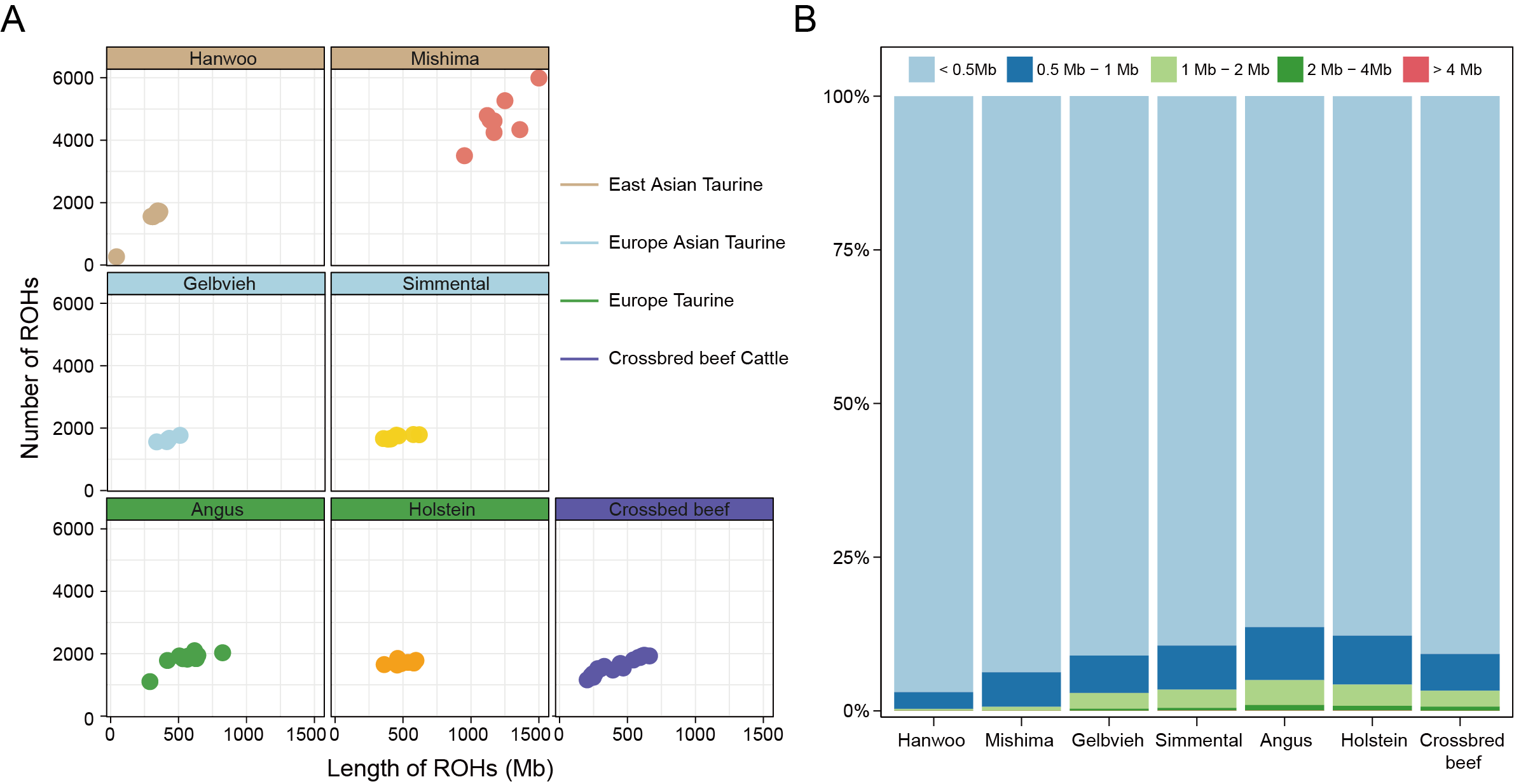

Supplement: Supplementary file 3 [file Image2.JPEG]
