# Supplementary material for: Identification of Heilongjiang crossbred beef cattle pedigrees and reveals functional genes related to economic traits based on whole-genome SNP data
Source: Front Genet. 2024 Jul 25;15:1435793. doi: 10.3389/fgene.2024.1435793 (PMC11306169; doi:10.3389/fgene.2024.1435793)
Supplement: Supplementary file 4 [file Table1.DOC]

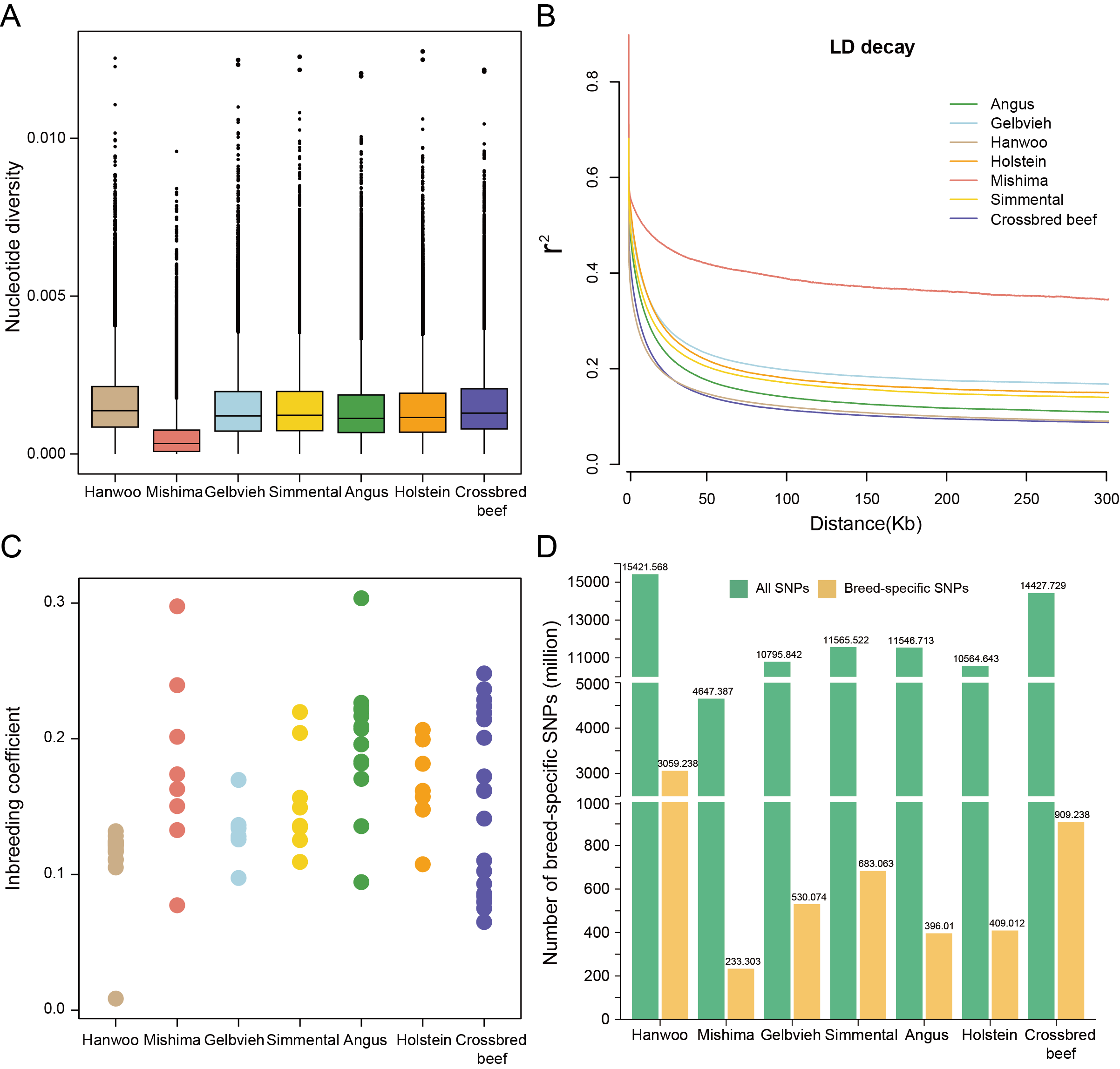


**Supplementary Figure S1** Genetic diversity among 7 breeds.

(A) Box plots of the nucleotide diversity for each breed. The points which were on the outside of the whiskers showed outliers. (B) Decay of linkage disequilibrium on cattle autosomes estimated from each breed. (C) Inbreeding coefficient for each breed. (D) Number of SNPs for each breed.
