# Supplementary material for: Identification of Heilongjiang crossbred beef cattle pedigrees and reveals functional genes related to economic traits based on whole-genome SNP data
Source: Front Genet. 2024 Jul 25;15:1435793. doi: 10.3389/fgene.2024.1435793 (PMC11306169; doi:10.3389/fgene.2024.1435793)
Supplement: Supplementary file 5 [file Table2.DOC]

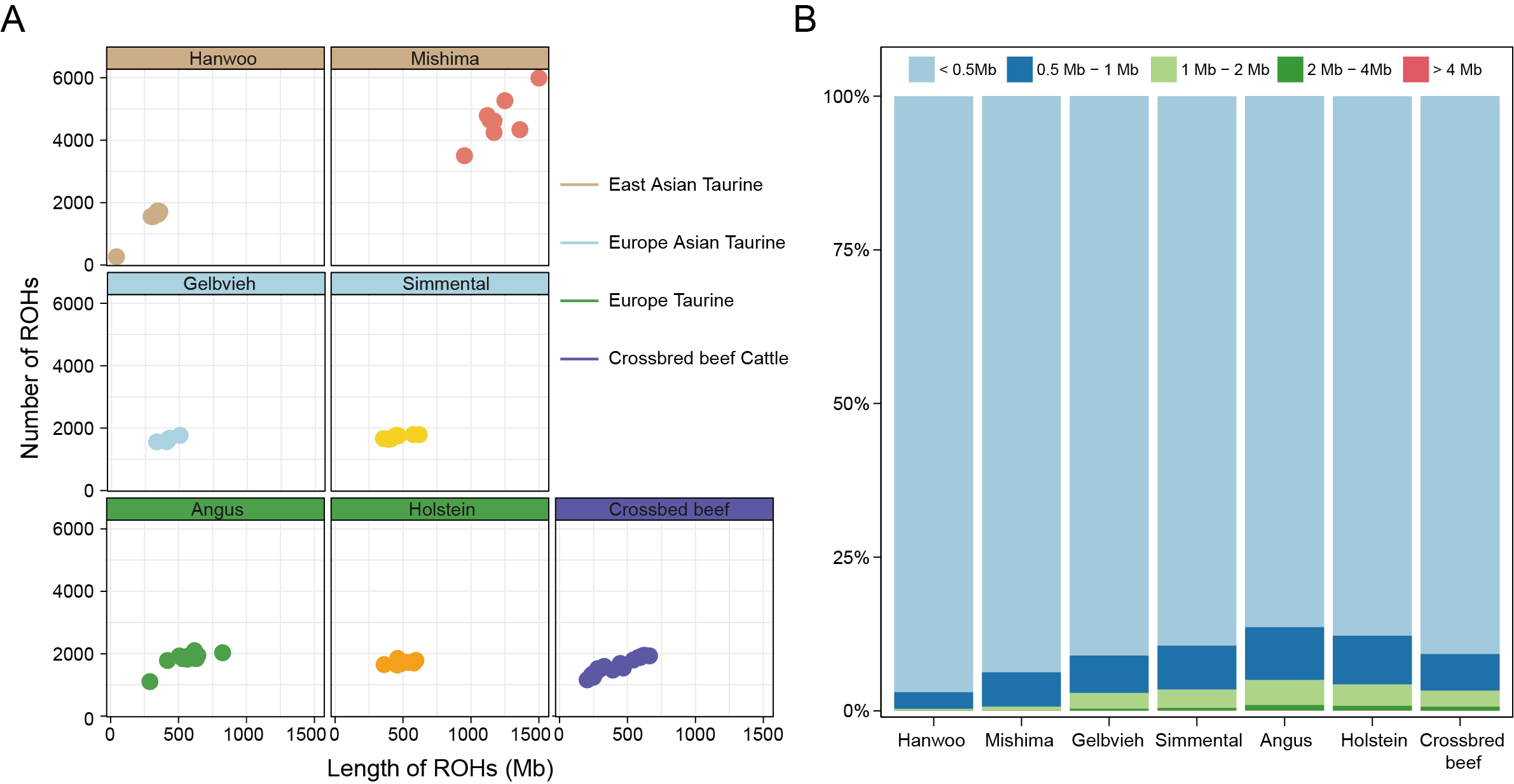


**Supplementary Figure S2** ROH analysis among 7 breeds.

(A) Total length and total number of ROHs per individual in each breed. (B) The proportion of different categories of ROHs in each breed.
